# Correlative and Live-Cell Imaging of S100B-Promoter-Associated Interstitial Cells from the Cardiac Sinoatrial Node and Valves

**DOI:** 10.3390/cells15191762

**Published:** 2026-09-27

**Authors:** Dmitry B. Zorov, Miguel Calvo-Rubio, Robert E. Monticone, Bruce D. Ziman, Richard Telljohann, Georgiana Luisa Baca, Khalid Chakir, Valeriya B. Vays, Irina M. Vangeli, Lora E. Bakeeva, Ljubava D. Zorova, Rostislav Bychkov, Edward G. Lakatta

**Affiliations:** 1 Laboratory of Cardiovascular Science, Intramural Research Program, National Institute on Aging, National Institutes of Health, Baltimore, MD 21224, USA; 2 A.N. Belozersky Institute of Physico-Chemical Biology, M.V. Lomonosov Moscow State University, 119992 Moscow, Russia; 3 Translational Gerontology Branch, National Institute on Aging, National Institutes of Health, Baltimore, MD 21224, USA

**Keywords:** telocytes, sinoatrial node, cardiac valves, S100B-EGFP, interstitial cells, mitochondria, NAD(P)H, mitochondrial membrane-potential-sensitive fluorescence, correlative light and electron microscopy, live imaging, cytoplasmic fragments, nuclear heterogeneity

## Abstract

Cardiac pacemaker and valve cells operate within a heterogeneous interstitial environment, but the identities and observable behaviors of many constituent cells remain incompletely defined. We integrated complementary imaging observations from a single experimental platform into an exploratory analysis of cultured cells isolated from the adult mouse sinoatrial node (SAN) and atrioventricular valves of S100B-EGFP reporter mice. Cellular morphology, endogenous NAD(P)H fluorescence, tetramethylrhodamine methyl ester (TMRM) fluorescence, correlative light and electron microscopy (CLEM), time-lapse imaging, transmission electron microscopy (TEM), and 5-ethynyl-2′-deoxyuridine (EdU) labeling were used to describe selected reporter-positive and reporter-negative cells. S100B-promoter-associated EGFP+ cells displayed heterogeneous forms, including cells with small bodies and long processes. In representative fields, EGFP+ cells showed greater NAD(P)H autofluorescence intensity and lower TMRM signal than adjacent EGFP− cells. CLEM related selected fluorescence phenotypes to ultrastructure in the same cells and documented mitochondrial, vesicular, nuclear, and membrane-associated profiles. One live sequence captured movement of a TMRM-positive, mitochondrion-like structure through a thin intercellular bridge toward an EGFP− cell, followed by bridge disassembly. Additional images showed mitochondrial and vesicular profiles near cell surfaces or in extracellularly situated regions, elongated NAD(P)H-bright structures, reporter-positive cytoplasmic fragments, nuclear size heterogeneity, a narrow connection between nuclear profiles, and unequal EdU labeling among nuclear fragments. These findings are descriptive and hypothesis-generating. They do not establish definitive cell identity, phenotype prevalence, active or selective secretion, tunneling-nanotube identity, mitochondrial metabolic competence, regulated nuclear remodeling, recipient-cell uptake, or physiological consequence. The study provides an integrated morphological framework and a set of candidate events for future quantitative investigation of S100B-associated cardiac interstitial-cell biology.

## 1. Introduction

The heart is often described through its excitable and contractile cells, yet those cells function within a multicellular tissue architecture. Human cardiac atlases identify extensive diversity among cardiomyocytes, fibroblasts, endothelial cells, pericytes, smooth muscle cells, immune cells, neuronal cells, and other populations [1,2]. In the SAN, heterogeneous local and subthreshold Ca^2+^ signals arising within and among cells self-organize into synchronized, tissue-level impulses [3]. This emergent behavior makes the surrounding cellular environment relevant to rhythm generation without assigning control to any single interstitial population.

Prior intact-tissue studies provide the anatomical and physiological context for the present work. In ex vivo mouse SAN, Bychkov et al. described S100B+/GFAP− interstitial cells with heterogeneous cell bodies and long processes within the HCN4+ pacemaker-cell meshwork, together with a structurally distinct telocyte population identified by TEM near pacemaker cells, neuronal fibers, and capillaries [4]. Correspondence between the S100B+ cells and TEM-defined telocytes was supported by morphology and location but was not definitively established because S100B was not labeled at electron-microscopic resolution. The same study reported that superfusion with purified human S100B markedly altered local Ca^2+^ dynamics and action-potential firing rate and rhythm, with much of the action-potential-interval effect reversing after washout [4]. Human studies have independently described telocytes or telocyte-associated interstitial cells in the SAN and in mitral, tricuspid, and aortic valves [5,6]. These observations justify examining SAN- and valve-derived interstitial cells, but they do not establish that cultured reporter-positive cells are identical to any in situ population.

The broader SAN microenvironment may also be functionally relevant through pathways unrelated to S100B. For example, CCK/CCKAR signaling involving SAN fibroblasts, intrinsic cardiac neurons, and pacemaker cardiomyocytes can alter pacemaker-cell automaticity in defined experimental settings [7,8]. Such work supports investigation of non-myocyte populations while underscoring the need to distinguish anatomical proximity and reporter expression from demonstrated function.

In the transgenic mice used here, EGFP expression is driven by the human S100B promoter [9]. Reporter fluorescence therefore identifies cells with promoter activity, but is not a direct measurement of S100B protein abundance, release, or subcellular localization. Telocytes are generally recognized through a combination of tissue location, a small cell body, long thin telopodes with focal dilations, ultrastructure, relationships with neighboring structures, and context-dependent phenotypic markers [10,11,12,13,14,15,16]. No single protein marker uniquely identifies telocytes across organs [17]. Because the present cultured cells lack validated in situ localization and a definitive molecular marker panel, we use the neutral terms S100B-EGFP+ interstitial cells, reporter-positive interstitial cells, or S100B-promoter-associated cells. The term telocyte is reserved for cells identified as such in the cited tissue literature.

Mitochondrial organization and redox-associated fluorescence offer a descriptive view of cellular phenotype. Mitochondria participate in biosynthesis, signaling, redox regulation, differentiation, and cell death as well as ATP production [18,19,20,21,22,23,24,25,26]. Endogenous NAD(P)H fluorescence can be recorded without an added probe, but conventional intensity imaging cannot distinguish NADH from NADPH and is influenced by cofactor pool size, reduction state, protein binding, cell geometry, and optical conditions [27,28,29,30,31,32]. Fluorescent probe-based potentiometric signals likewise depend on dye concentration, binding, quenching, plasma-membrane potential, mitochondrial volume, cell geometry, and optical dilution [33,34,35,36,37,38,39,40]. These readouts are therefore treated here as imaging phenotypes rather than direct measurements of respiration, ATP synthesis, oxidative-stress resistance, or absolute mitochondrial membrane potential.

Intercellular movement of mitochondria and other cellular material has been reported through thin connections, extracellular vesicles, extracellular release, and experimental transplantation in multiple systems [41,42,43,44,45,46,47,48,49,50,51,52,53,54,55,56,57,58,59,60,61,62,63,64,65,66,67,68,69,70,71,72,73,74,75,76]. However, recent nomenclature recommendations emphasize that organelle identity, route, directionality, persistence, and recipient-cell effects require independent validation [53]. Correlative microscopy further shows that structures with similar light-microscopic appearances may have distinct ultrastructural organizations, so bridge morphology alone does not establish a tunneling nanotube [77]. CLEM can nevertheless be valuable for linking fluorescence-defined cells or rare events to ultrastructure [78,79].

The present study integrates complementary observations generated within the same experimental platform. Its aims were to: (i) describe reporter-positive cell morphology in SAN- and valve-derived cultures; (ii) document representative NAD(P)H and TMRM fluorescence patterns; (iii) relate selected fluorescence phenotypes to ultrastructure by CLEM; (iv) present one live-imaged movement event through a thin intercellular bridge; and (v) catalogue additional mitochondrial, cytoplasmic, and nuclear profiles that generate testable hypotheses. The multicellular adult mouse SAN culture platform is described elsewhere [80].

## 2. Materials and Methods

### 2.1. Study Design and Interpretive Scope

This was an exploratory, descriptive imaging study. Selected reporter-positive and reporter-negative cells were examined by confocal microscopy, correlative light and electron microscopy (CLEM), time-lapse imaging, transmission electron microscopy (TEM), and EdU labeling. S100B-EGFP fluorescence was treated as a marker of S100B-promoter activity rather than direct quantification or localization of S100B protein. Fluorescence ratios were summarized descriptively at the independent-preparation level as specified below; no inferential statistical comparisons were performed.

### 2.2. Mice

B6;D2-Tg(S100B-EGFP)1Wjt/J mice were purchased from The Jackson Laboratory (Bar Harbor, ME, USA). In these mice, EGFP expression is under control of the human S100 calcium-binding protein promoter [9]. All studies were performed in accordance with the Guide for the Care and Use of Laboratory Animals published by the National Institutes of Health (NIH Publication no. 85-23, revised 1996). Experimental protocols were approved by the NIH Animal Care and Use Committee (protocol no. 457-LCS-2027). A total of 22 mice were used: 18 mice contributed 18 independent SAN cell preparations, and four different mice contributed four independent valve cell preparations. A total of 22 mice were used: 18 mice contributed 18 independent SAN cell preparations (16 males and 2 females of age 18–66 weeks), and 4 different mice contributed 4 independent valve cell preparations (3 males and 1 female of age 18–28 weeks).

### 2.3. Mouse SAN and Cardiac-Valve Cell Isolation

Mice were euthanized with pentobarbital. Hearts were removed and placed in cold phosphate-buffered saline. The right atrium was removed, and SAN tissue was cut into strips perpendicular to the crista terminalis. The remaining heart was cut laterally through the left and right ventricles at the atrioventricular ring to expose the atrioventricular valves, which were removed by cutting around the hinge region. SAN and valve tissues were processed with the same digestion protocol. The general SAN-cell isolation approach followed prior work [81].

Tissues were placed in warm Tyrode solution containing (in mmol/L) 140 NaCl, 5.4 KCl, 0.5 MgCl_2_, 0.33 NaH_2_PO_4_, 5 HEPES, and 5.5 glucose (pH 7.0) for 10 min at 35 degrees C. They were then digested in 3 mL Ca^2+^-free Tyrode solution containing lyophilized elastase (2.0 mg; Worthington Biochemical, Lakewood, NJ, USA), protease (0.4 mg; Sigma- Aldrich, Burlington, MA, USA), collagenase type II (3 mg; Worthington Biochemical), and bovine serum albumin (3 mg; Sigma) for 30 min at 35 degrees C with gentle shaking. Tissue was transferred to 2 mL modified KB solution containing 70 potassium glutamate, 30 KCl, 10 KH_2_PO_4_, 1 MgCl_2_, 20 taurine, 10 glucose, 0.3 EGTA, and 10 HEPES (pH 7.3 adjusted with KOH) at 4 degrees C for 20 min. Tissue was then transferred to 200–300 microliters fresh KB solution at room temperature, minced, and dispersed by gentle pipetting.

### 2.4. Cell Culture

Valve and SAN cells (50,000–100,000) were centrifuged at 200× *g* for 5 min, pelleted, and resuspended in 200–300 microliters growth medium. Valve cells were first plated in one well of a collagen-coated 6-well dish and allowed to proliferate. Near confluence, they were trypsinized and replated at 30,000–50,000 cells in 200–300 microliters onto the glass center of collagen-coated 35-mm dishes (P35GCOL-1.0-14C; MatTek, Gainsville, FL, USA). SAN cells were plated directly onto comparable glass-bottom dishes. Growth medium consisted of DMEM/F12 (1:1; GIBCO, Waltham, MA, USA) supplemented with MEM non-essential amino acids (GIBCO), Pen-Strep (GIBCO), and 10% fetal bovine serum (R&D Systems, Minneapolis, MN, USA). Cultures were examined after 2–3 days or 4–5 days after plating, depending on the experiment [80]. Because valve-derived cells underwent expansion, trypsinization, and replating, whereas SAN-derived cells were plated directly, tissue-source effects cannot be separated from culture-history effects; direct SAN-versus-valve phenotype comparisons were therefore not performed.

### 2.5. Confocal and Live-Cell Microscopy

Dishes were imaged with an LSM 880 laser-scanning confocal microscope (Carl Zeiss, Jena, Germany). NAD(P)H autofluorescence was excited at 355 nm and EGFP at 488 nm; emissions were collected at 371–487 nm and 490–553 nm, respectively. Objective: Plan-Apochromate 63×/1.4 Oil DIC M27. Image size: 1024 × 1024 pixels. The pinhole was set at 5.55 AU. Mitochondrial-associated signals were visualized either through NAD(P)H autofluorescence or after staining with tetramethylrhodamine methyl ester (TMRM; final bath concentration 500 nM for 20 min), followed by excitation at 561 nm and emission collection at 563–735 nm. Separate control cultures were exposed to 100 nM FCCP or 5 µM nigericin to assess loss of TMRM fluorescence after mitochondrial de-energization. Mitochondrial morphology was interpreted descriptively in light of the broader literature on mitochondrial dynamics [82].

For time-lapse imaging, the same channels were recorded repeatedly in living cultured cells. The displayed sequence contains 10 time points from 0 to 45 min. The Supplementary Video S1 provides the full recording.

### 2.6. Correlative Light and Electron Microscopy and TEM

For CLEM, cells were seeded onto gridded glass-bottom Petri dishes. Selected cells were imaged by confocal microscopy and localized on the grid. They were fixed in 2.5% glutaraldehyde and 2% paraformaldehyde in 0.1 M sodium cacodylate buffer (pH 7.2) for 12–24 h, washed, and post-fixed in 1% osmium tetroxide for 1 h at 4 degrees C. Samples were dehydrated through an ascending ethanol series, transferred to propylene oxide, and infiltrated with EMbed 812 resin (Electron Microscopy Sciences, Hatfield, PA, USA) using propylene oxide:resin mixtures of 2:1, 1:1, and 1:2 over 24 h (8 h each), followed by pure resin for 24 h and polymerization for 48 h at 65 degrees C. Glass was removed by alternating transfers between liquid nitrogen and hot water. Blocks were sectioned with an Ultracut Reichert or Leica (Wetzlar, Germany) ultramicrotome. Thin sections (40–70 nm) were mounted on nickel grids and stained with uranyl acetate and lead citrate. Images were acquired with a JEM1400 electron microscope (JEOL, Tokyo, Japan) equipped with a QUEMESA camera (Olympus, Center Valley, PA, USA).

Some TEM images were acquired without same-cell correlation to reporter fluorescence. Those images are therefore presented as observations from the cultured preparation without assigning reporter status or cell type at electron-microscopic resolution. Static two-dimensional profiles were not interpreted as proof of secretion, uptake, division, degradation, or directionality.

### 2.7. EdU Labeling

DNA synthesis was assessed by incorporation of 5-ethynyl-2′-deoxyuridine (EdU), followed by reaction with a fluorescent azide in a copper-catalyzed [3 + 2] cycloaddition. Labeling used the Click-iT EdU Imaging Kit (Invitrogen, Carlsbad, CA, USA) according to the manufacturer’s instructions. Glass-bottom dishes were fixed with 4% paraformaldehyde in phosphate-buffered saline for 15 min, washed twice with 3% bovine serum albumin in phosphate-buffered saline, permeabilized with 0.5% Triton X-100 in phosphate-buffered saline for 20 min, washed twice, and incubated with Click-iT reaction cocktail containing reaction buffer, CuSO_4_, Alexa Fluor 594 azide, and reaction-buffer additive for 30 min protected from light. All steps were performed at room temperature. EdU signal was treated as evidence of DNA synthesis; the images do not distinguish replication from DNA repair or establish when nuclear fragmentation occurred relative to labeling.

### 2.8. Image Presentation and Data Analysis

Related observations were organized into composite figures. The original image files were retained, and group labels were added for the integrated layout. Scale bars, arrows, and internal panel labels present in the original images were preserved. Results from CLEM, TEM, time-lapse imaging, and EdU labeling refer to the displayed fields and selected cells and are not estimates of population prevalence.

Fluorescence intensity was evaluated in three ways: (1) whole-cell integrated intensity; (2) cytoplasmic intensity measured in nine regions selected away from the nucleus; and (3) intensity measured across nine individual mitochondrial filaments per cell. The nine regions or filaments were treated as technical subsamples and were not considered independent biological replicates. Within each preparation, measurements from selected EGFP+ and adjacent EGFP− cells were aggregated to one paired fluorescence ratio. For statistical analysis, totally, 10 EGFP+ cells and 13 EGFP− cells for TMRM and NAD(P)H fluorescence intensities and transmittance were used (note that in 3 preps, 1 EGFP+ cell was compared with 2 EGFP− adjacent cells on the same prep). Each independent preparation originated from a different mouse and was the biological unit. Ratios were expressed as fluorescence intensities EGFP+/EGFP− for NAD(P)H and EGFP−/EGFP+ for TMRM, preserving the direction used in the source analysis. Values are reported as mean ± SEM. The standard error was estimated based on the averaged values for each preparation (n = 7 and 10). The exact number of independent preparations/cells for each metric was: NAD(P)H whole cell = 10; NAD(P)H peripheral cytoplasm = 7; NAD(P)H mitochondrial filaments = 7; TMRM whole cell = 10; TMRM peripheral cytoplasm = 7; TMRM mitochondrial filaments = 7. No inferential statistical comparisons were performed.

## 3. Results

### 3.1. Morphological Heterogeneity of S100B-Promoter-Associated Interstitial Cells

Reporter-positive cells in SAN- and valve-derived cultures displayed heterogeneous forms. Some had a small cell body and long, branching processes with focal swellings, whereas others had a broader cell body and shorter processes (Figure 1a). A second field showed a compact reporter-positive cell with extended processes and a mitochondrial network visible by NAD(P)H autofluorescence (Figure 1b). The displayed SAN- and valve-derived cells showed broadly similar forms; however, because the two sources underwent different post-isolation culture histories, this observation was not treated as a direct source-to-source phenotype comparison. These images describe morphology only and do not establish telocyte, fibroblast, glial, or other lineage identity.

### 3.2. Representative Mitochondrial and Redox-Associated Fluorescence Patterns

In selected reporter-positive cells, EGFP fluorescence did not visibly occupy the mitochondrial territory identified by NAD(P)H autofluorescence (Figure 2a). This separation was observed in cells with short mitochondrial profiles and in cells with a branched mitochondrial reticulum. Because EGFP is driven by the S100B promoter, the observation concerns reporter distribution and does not localize endogenous S100B protein.

In the representative SAN field shown in Figure 2b, the central EGFP+ cell had greater NAD(P)H autofluorescence intensity than adjacent EGFP− cells and a lower TMRM signal. Preparation-level ratios were calculated as described in the Methods. For NAD(P)H, the EGFP+/EGFP− ratio was 170 ± 6% for whole-cell integrated intensity, 147 ± 10% for peripheral cytoplasmic intensity, and 165 ± 11% for mitochondrial-filament intensity. For TMRM, the EGFP−/EGFP+ ratio was 219 ± 26%, 191 ± 21%, and 171 ± 14%, respectively. Values are mean ± SEM; The combined NAD(P)H and TMRM fluorescence phenotype distinguishes the selected EGFP+ and EGFP− cells under the present imaging conditions. However, because both signals are influenced by multiple metabolic, structural, and optical determinants, these measurements do not establish differences in respiratory activity, ATP production, oxidative-stress resistance, or absolute mitochondrial membrane potential.

In separate control images, TMRM fluorescence over mitochondrial-appearing profiles was lost after mitochondrial de-energization with 100 nM FCCP or 5 µM nigericin which acts in this concentration as a uncoupler [83]. A nigericin example is presented in Supplementary Figure S1. These controls confirm that the signal was responsive to mitochondrial de-energization under the imaging conditions; they do not calibrate absolute membrane potential or exclude other determinants of fluorescence intensity.

### 3.3. Thin Intercellular Contacts and Reporter-Positive Tethered Material

Reporter-positive cells contacted neighboring reporter-negative cells through thin processes and close membrane appositions (Figure 3). A TMRM-positive profile was visible within one thin bridge in a valve-derived culture (Figure 3a). In another field, EGFP fluorescence remained visually confined to the reporter-positive cell across the contact site (Figure 3b). This absence of detectable EGFP spread does not exclude limited cytoplasmic continuity or transfer of unlabeled material. A separate field showed adjacent reporter-positive and reporter-negative cells and a reporter-positive cytoplasmic projection or fragment tethered by thin connections (Figure 3c).

### 3.4. Same-Cell CLEM of Selected Reporter-Positive Cells

CLEM was used to relate reporter fluorescence to ultrastructure in selected cells. A confocal field on a gridded substrate contained a strongly reporter-positive cell, a cell with predominantly nuclear reporter fluorescence, a reporter-negative cell, and cellular remnants used for navigation (Figure 4a). Semi-thin and ultrathin sections were then used to locate the same reporter-positive cell and reconstruct its overall ultrastructure (Figure 4b). This workflow provided same-cell correlation but was not a population-sampling design.

Higher-magnification sections of the CLEM-mapped reporter-positive cell showed mitochondrial and vesicular profiles near the cell surface, membrane-bounded spaces containing cellular material, mitochondrial profiles with altered morphology, two nuclear profiles of different sizes, and an extended branched mitochondrial reticulum in the main cell body (Figure 5). A narrow opening to the extracellular space was visible in one ultrathin section. Because these are static two-dimensional sections, they cannot determine whether material was released, internalized, degraded, undergoing division, or intersected tangentially by the section plane.

### 3.5. Pairwise Ultrastructural Comparison and an Elongated Reporter-Positive Cell

A selected reporter-negative cell from the CLEM field was reconstructed and examined in contact with a neighboring cell showing nuclear EGFP fluorescence (Figure 6a). Within this selected pair, Golgi cisternae and rough endoplasmic reticulum were prominent in the reporter-positive cell, whereas rough endoplasmic reticulum predominated in the reporter-negative cell. Mitochondrial configuration also appeared different: profiles in the reporter-positive cell were generally smaller, with more densely packed cristae and a more condensed matrix, whereas the reporter-negative cell showed more autophagic profiles and a more prominent cytoskeleton. These are observations from one selected pair; the identity of the reporter-negative cell is unknown, and the differences cannot be generalized to cell populations.

In a separate SAN experiment, an unusually elongated reporter-positive cell was imaged by EGFP, NAD(P)H, TMRM, transmitted light, and serial TEM reconstruction (Figure 6b). Elongated mitochondrial profiles were visible along its long axis. The reconstruction documents cell shape and organelle distribution but does not establish specialized donor function.

### 3.6. A Single Live-Imaged Movement Event Through a Thin Intercellular Bridge

Static images raised the possibility that cellular material could move through thin contacts, but they could not establish directionality. In one live recording, an S100B-EGFP+ cell contacted an EGFP− cell through a narrow bridge containing a TMRM-positive, mitochondrion-like structure (Figure 7a). Across 10 displayed time points from 0 to 45 min, the structure moved toward the EGFP− cell along the bridge (Figure 7b). The bridge subsequently began to disassemble 35 min after the structure reached the neighboring cell (Figure 7c). We describe this as a putative mitochondrial-movement or transfer event. TMRM fluorescence is consistent with a polarized mitochondrion-like structure, but the recording does not independently establish organelle identity, complete incorporation, persistence, directionality as a general property, energetic competence, or a functional effect on the neighboring cell.

### 3.7. Static TEM Profiles near Cell Surfaces and in Extracellularly Situated Regions

Additional TEM images showed mitochondrial profiles attached near the plasma membrane, mitochondrial-appearing profiles in extracellularly situated regions, and membrane-bounded or invagination-like profiles containing mitochondria and vesicles (Figure 8).

### 3.8. Elongated NAD(P)H-Bright Structures Extending from Reporter-Positive Cells

Two confocal fields showed elongated NAD(P)H-bright structures extending from regions of S100B-EGFP+ cells (Figure 9). The structures did not show detectable EGFP fluorescence in the displayed channels. Their morphology and endogenous fluorescence are compatible with mitochondrial material.

### 3.9. Nuclear Heterogeneity and EdU Labeling

Serial ultrathin sections of one selected cell showed two nuclear profiles connected by a narrow bridge (Figure 10a). A separate confocal field showed a small NucBlue-positive nuclear profile adjacent to a larger nucleus in an EGFP+ cell (Figure 10b). In another experiment, EdU signal varied among nuclear fragments in two reporter-positive cells (Figure 10c). These observations document nuclear heterogeneity and DNA synthesis-associated fluorescence. They do not establish active or regulated nuclear remodeling, asymmetric division, micronucleus formation after EdU incorporation, or exclusion of DNA repair, stress, degeneration, or cell damage. Nuclear-fragment phenomena have been studied extensively in cancer and cell-biological contexts [84,85,86,87,88,89,90,91,92].

### 3.10. Reporter-Positive Cytoplasmic Fragments and Cell-Overlying Configurations

Reporter-positive cells and fragments were sometimes positioned over reporter-negative cells (Figure 11a). Other fields showed reporter-positive cytoplasmic material connected to a larger cell by a narrow bridge, adjacent reporter-positive vesicular profiles, and detached reporter-positive fragments of varied shape (Figure 11b,c). Some fragments contained detectable NAD(P)H- or TMRM-associated fluorescence, with qualitatively low signal in selected examples. The fragments could reflect normal partitioning, culture-induced stress, cell damage, or degeneration.

## 4. Discussion

This study organizes complementary observations around one evidentiary sequence: reporter-defined morphology, mitochondrial and redox-associated fluorescence, same-cell ultrastructure, one live movement event, and additional static observations of mitochondrial, cytoplasmic, and nuclear profiles. The results do not indicate that a defined cardiac interstitial-cell type routinely acts as an organelle donor or structural signaling unit.

The intact mouse and human tissue literature supports the presence of telocytes in the SAN and cardiac valves and suggests correspondence between S100B+ interstitial cells and telocytes in mouse SAN [4,5,6]. In the present cultures, however, promoter-driven EGFP, variable morphology, loss of in situ location, and absence of a validated marker or transcriptomic panel prevent definitive classification. Reporter-negative cells are also unidentified. The data therefore advance knowledge of an S100B-promoter-associated interstitial compartment, not telocyte physiology as a specifically established cell type.

The inclusion of valve-derived cells is anatomical and comparative rather than mechanistic. Telocytes have been reported in human heart valves [6]. SAN and valve tissues were digested with the same protocol, but their post-isolation culture histories differed: valve-derived cells were expanded, trypsinized, and replated, whereas SAN-derived cells were plated directly. Consequently, the displayed similarities cannot distinguish tissue-origin effects from culture-history effects, and direct SAN-versus-valve phenotype comparisons were not performed.

The fluorescence observations define candidate phenotypes. Greater NAD(P)H autofluorescence intensity can reflect cofactor pool size, reduction state, binding, geometry, or several factors together [27,28,29,30,31,32]. TMRM intensity is similarly affected by probe behavior, plasma-membrane potential, mitochondrial mass and volume, cell shape, and optical dilution [33,34,35,36,37,38,39,40]. The preparation-level ratios therefore describe fluorescence differences under the present imaging conditions but do not establish lower respiration, ATP production, oxidative-stress resistance, or absolute mitochondrial membrane potential. Although high protonic potential can promote mitochondrial reactive-oxygen-species production in some systems [93], no oxidative-stress measurement was performed here.

CLEM strengthens the study by connecting fluorescence-defined cells with ultrastructure in the same field [78,79]. It also defines a strict boundary: the detailed CLEM observations apply to selected cells, not to a population. Peripheral mitochondrial profiles, membrane-bounded cavities, vesicles, and altered organelle morphology are compatible with several processes. Static sections cannot distinguish release from uptake, degradation, autophagy, damage, or tangential sectioning. The additional non-correlative TEM material lacks same-cell reporter correlation, so its cellular identity is even more limited. Displaying those images at larger size and revising the legends improves independent assessment, but higher-resolution original micrographs and additional low-to-high magnification series would still be needed to satisfy a fully quantitative ultrastructural analysis.

The live sequence provides temporal information absent from static TEM. A TMRM-positive, mitochondrion-like structure moved through a thin bridge toward an EGFP− cell, and the bridge later disassembled. Similar organelle movement has been documented in other systems [42,43,44,45,46,47,48,49,64,72,84,85,86]. However, the observation was a single event, the bridge was not structurally classified, and the moving profile was not verified with an organelle-specific label. The data therefore do not establish routine mitochondrial transfer, complete uptake, persistence, or recipient-cell function.

The static TEM and extracellularly situated profiles invite several alternative interpretations. Extracellular mitochondria and mitochondrial material have been reported in blood and in vesicular or transplantation contexts [41,42,43,44,45,46,47,48,49,61,62,63,64,65,66,67,68,69,70,71,72,73,74,75], but the functionality of apparently cell-free mitochondria remains debated [76]. In the heart, cardiomyocytes can externalize dysfunctional mitochondrial material for macrophage clearance [94]. Cancer cells can also use mitochondrial transfer in immune evasion [65]. These precedents show that extracellular or transferred mitochondrial profiles do not uniquely imply beneficial donation. In the present cultures, cell stress, degeneration, quality-control pathways, or debris from dying cells remain plausible alternatives.

The nuclear and cytoplasmic-fragment observations broaden the morphological catalogue of material partitioning in the same reporter-associated compartment. Serial sections documented a narrow inter-nuclear connection, and EdU labeling differed among nuclear fragments. The literature on microvesicles, oncosomes, motile cell fragments, enucleated cells, micronuclear collapse, and nuclear-membrane integrity provides relevant descriptive context [84,85,86,87,88,89,90,91,92]. However, EdU can accompany DNA replication or repair, and the present images do not establish the temporal order of labeling and fragmentation.

Several limitations define the next experiments. The fluorescence analysis used selected cell pairs rather than a prospectively sampled population and should be confirmed in a blinded, prespecified design. The study also lacks event-frequency measurements, viability controls, organelle-specific labeling, recipient-cell functional assays, and quantitative three-dimensional reconstruction. The reporter is not a direct S100B-protein assay, and the negative cells are not identified. Cell sorting followed by transcriptomic and marker analysis would address identity. Replicated live imaging with cytoskeletal and membrane markers would test bridge classification and event directionality. Organelle-specific labels, extracellular-mitochondria assays, and recipient-cell physiological readouts would be required to establish transfer, persistence, or function.

The scientific significance is therefore qualitative. Cardiac impulses emerge within a heterogeneous multicellular SAN [3], exogenous S100B can alter SAN Ca^2+^ dynamics and rhythm in an intact preparation [4], and defined non-myocyte signaling can influence pacemaker automaticity [8]. Against that background, the combined study provides a practical imaging framework for locating S100B-promoter-associated interstitial cells and for recognizing candidate mitochondrial, cytoplasmic, and nuclear events that can be tested quantitatively. No direct clinical application follows from the present observations, but a more complete map of cardiac interstitial-cell phenotypes may ultimately help explain how local tissue architecture supports conduction-system resilience or contributes to dysfunction.

## 5. Conclusions

Cultured S100B-promoter-associated interstitial cells from the mouse SAN and atrioventricular valves displayed heterogeneous morphologies, representative differences in NAD(P)H and TMRM fluorescence, and varied mitochondrial, vesicular, cytoplasmic, and nuclear profiles. Same-cell CLEM linked selected reporter phenotypes to ultrastructure. A live sequence recorded movement of a TMRM-positive, mitochondrion-like structure through a transient intercellular bridge toward an EGFP− cell. The work should be viewed as a descriptive first step in quantitative investigation of S100B-associated cardiac interstitial-cell biology. Our observations define candidate phenomena for further study but do not establish definitive cell identity, active secretion, selective or routine mitochondrial transfer, regulated nuclear remodeling, recipient-cell uptake, or physiological function.

## Figures and Tables

**Figure 1:**
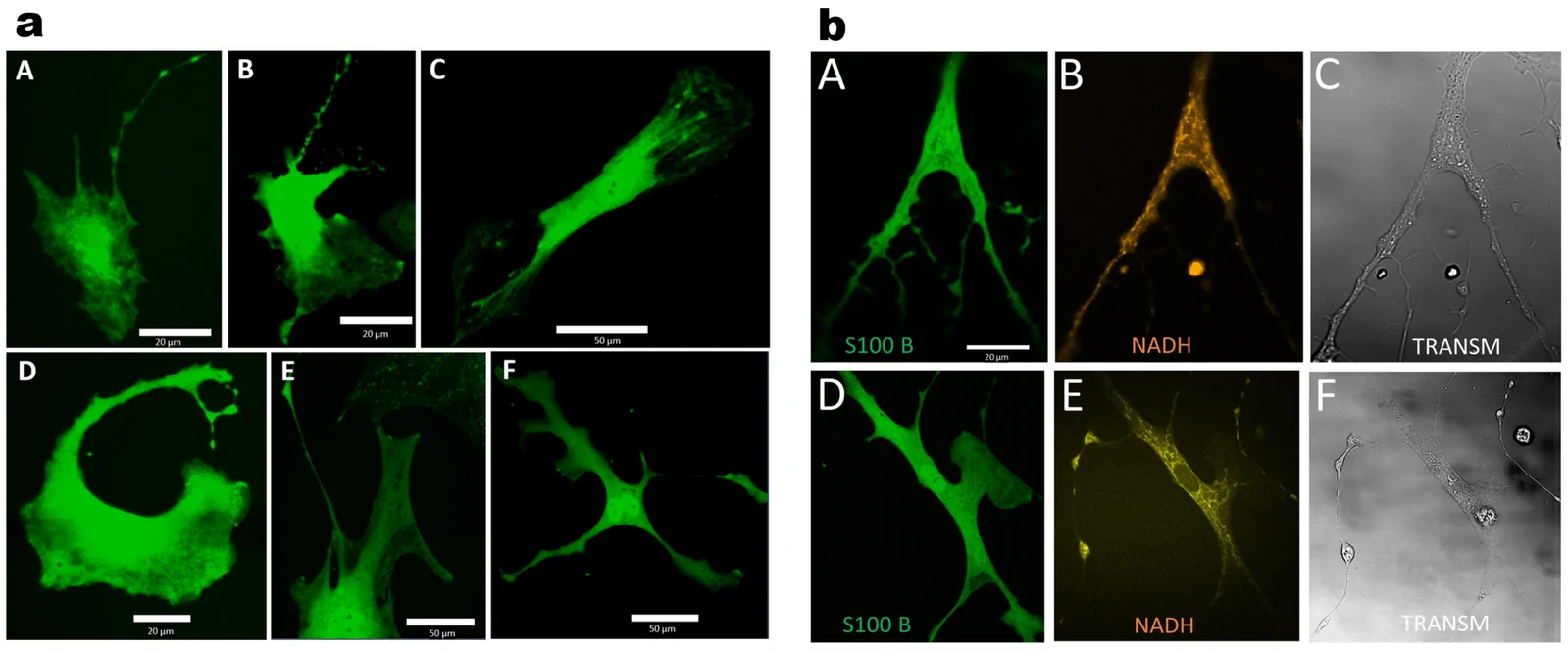
Morphological heterogeneity of S100B-promoter-associated EGFP+ interstitial cells in adherent culture. (**a**) Representative reporter-positive forms from SAN-derived cultures (original panels (A–D)) and atrioventricular-valve-derived cultures (original panels (E,F)). (**b**) A reporter-positive cell with extended processes shown with EGFP fluorescence (A,D), NAD(P)H autofluorescence (B,E), and transmitted light (C,F). Lowercase labels identify the grouped image sets; original panel labels and scale bars are retained.

**Figure 2:**
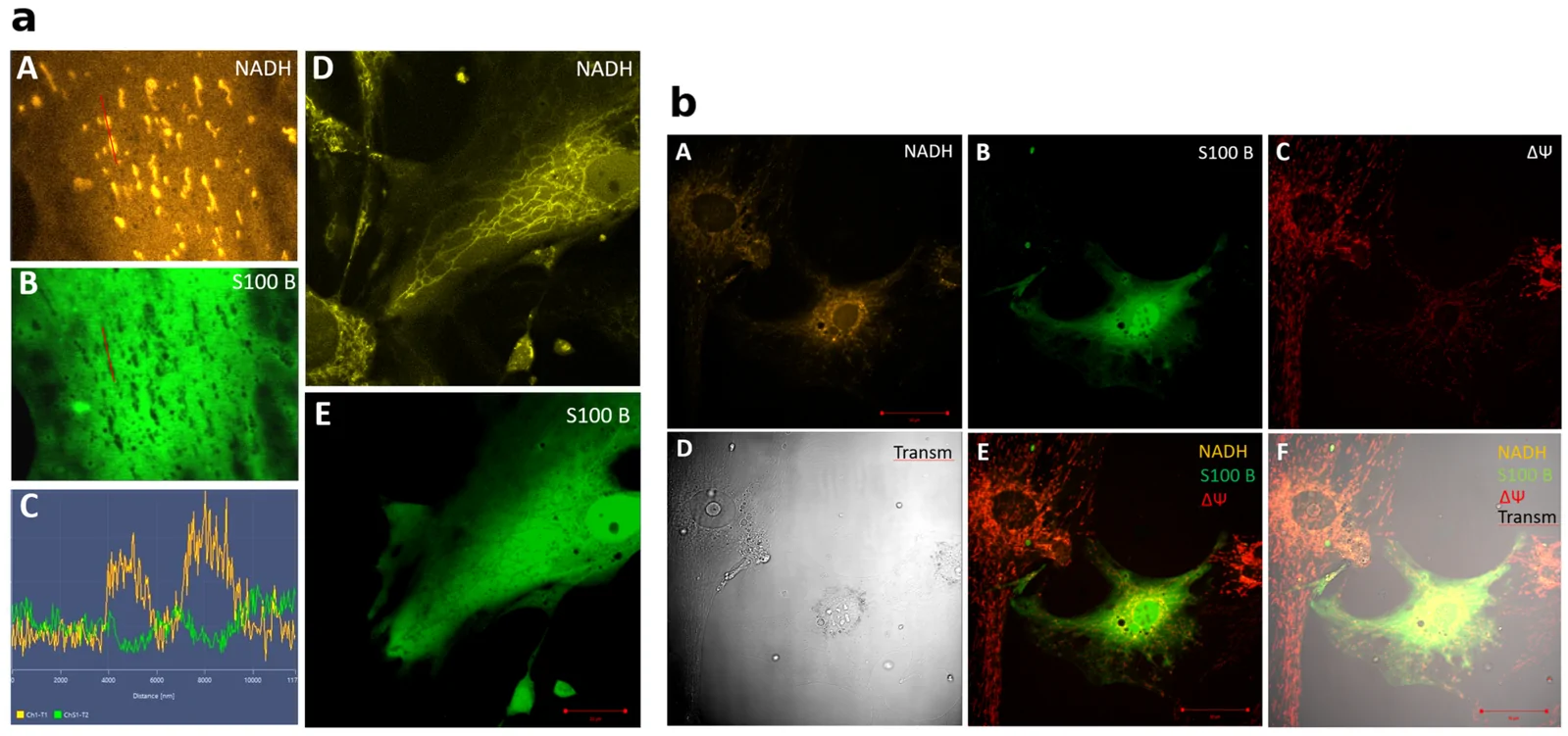
Representative fluorescence phenotypes. (**a**) Separation of S100B-promoter-associated EGFP fluorescence from the mitochondrial territory visualized by NAD(P)H autofluorescence in SAN-derived cells (original panels (A–C)) and valve-derived cells (original panels (D,E)). The source red line profile along mitochondria in A and B is retained and digitized in C (**b**) Representative SAN field containing an EGFP+ cell and adjacent EGFP− cells. The reporter-positive cell shows stronger NAD(P)H autofluorescence (A) and lower TMRM fluorescence in this field (C). B, D, E and F show fluorescence of the reporter cell, transmittens and overlays correspondingly. Original channel labels and scale bars are retained. These images are illustrative and do not constitute a population-level statistical comparison or direct measurement of respiration or absolute mitochondrial membrane potential.

**Figure 3:**
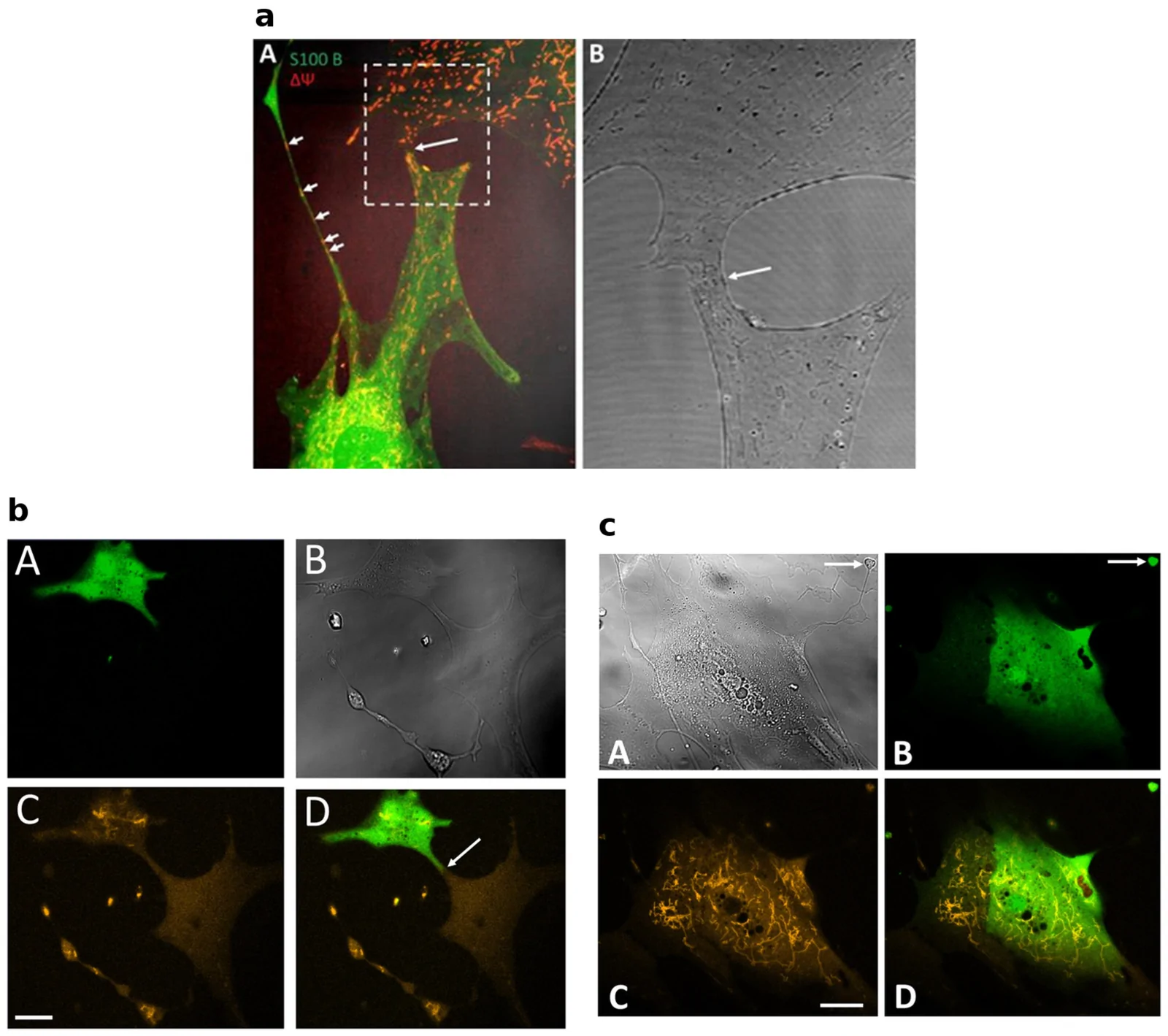
Thin contacts and tethered reporter-positive material. (**a**) A thin intercellular bridge in a valve-derived culture contains TMRM-positive profiles (short arrows) and connects an S100B-EGFP+ cell to a neighboring EGFP− cell. A, overlay of a reporter cell and TMRM fluorescence; B, transmittance. Transmittance image (B) shows tight interaction (long arrow in A and B) of two cells highlighted by the dashed box in A; (**b**) Direct contact between a reporter-positive and reporter-negative cell without detectable spread of EGFP fluorescence across the field. A, B, C and D, a reporter cell fluorescence, transmittance, NAD(P)H fluorescence and overlay, correspondingly; (**c**) Adjacent reporter-positive and reporter-negative cells with a reporter-positive projection or fragment retained by thin connections. A, B, C and D, transmittance, reporter cell fluorescence, NAD(P)H fluorescence and overlay, correspondingly. Arrow shows remote piece with green fluorescence (B) connected with mother cell by a long thin filament. Lowercase labels identify grouped image sets; original panel labels, arrows, and scale white bars (10 µm) are retained.

**Figure 4:**
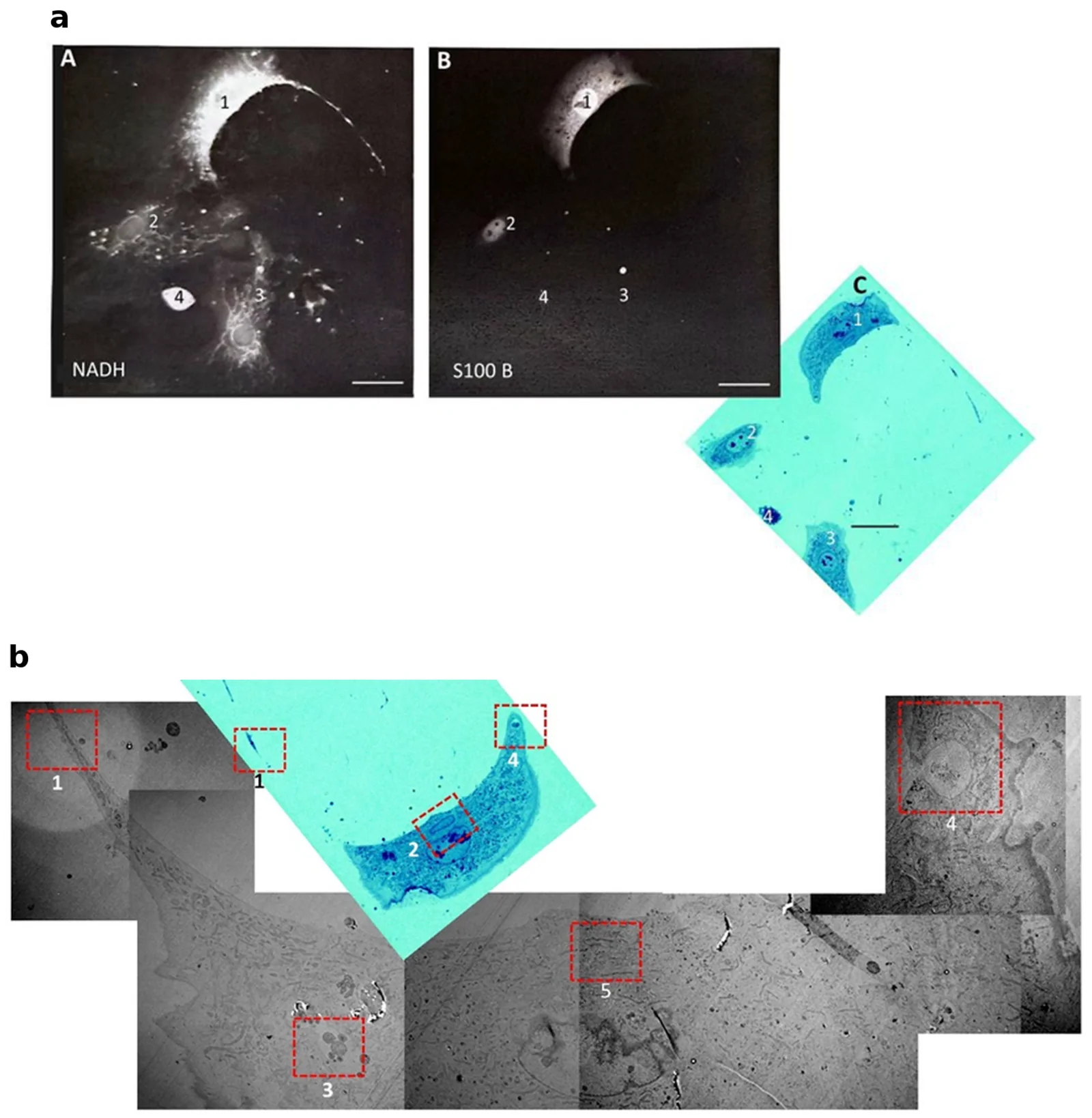
Same-field and same-cell CLEM workflow. (**a**) NAD(P)H (A) and S100B-promoter-associated EGFP fluorescence (B) in a gridded SAN culture, followed by a semi-thin section of the same region. C, Toluidine-Blue-staining. Object 1 is reporter-positive, object 2 has predominantly nuclear reporter fluorescence, object 3 is reporter-negative, and object 4 comprises cellular remnants used as navigation landmarks. (**b**) Low-magnification electron-microscopic reconstruction of object 1, with the Toluidine-Blue-stained semi-thin section superimposed and regions selected for higher-magnification examination. Lowercase labels identify grouped image sets; original object numbers, boxes, and scale bars are retained.

**Figure 5:**
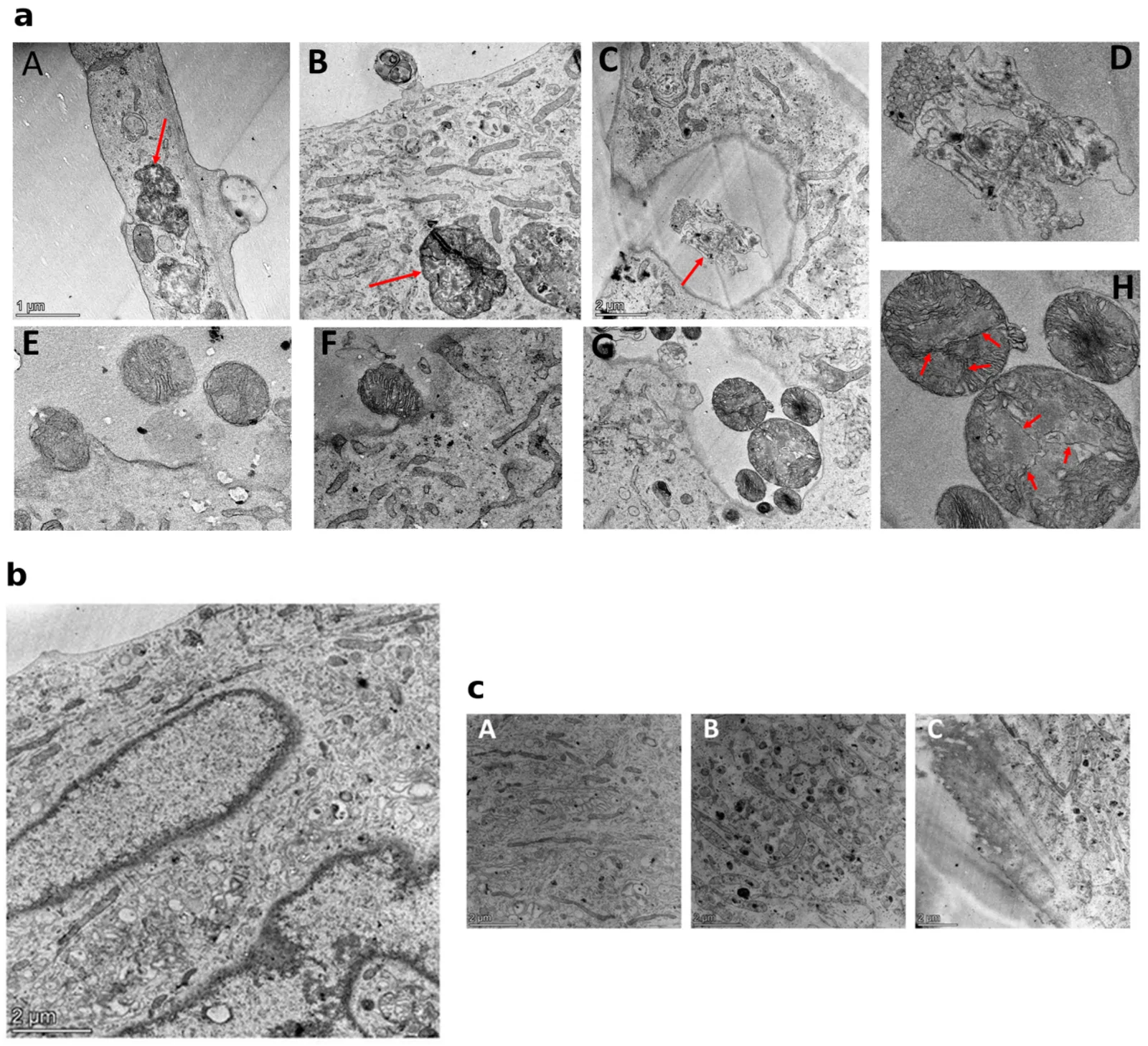
Ultrastructural details of the CLEM-mapped reporter-positive cell in Figure 4a,b. (**a**) A, B, E and F are selected high-magnification regions marked by square 1 in Figure 4b. C and D show a space apparently containing cellular components destined for release from the cell, including vesicles and damaged mitochondria (red arrows; D is a higher magnification). Note that this space appears closed on the semi-thin section (square 4 in Figure 4b). G and H show region 3 in Figure 4b at low and high resolution, respectively. (**b**) Ultrathin perinuclear section showing nuclear profiles of different sizes. (**c**) A–C, mitochondrial reticulum in the main cell body (from square 5 in Figure 4b), including extended and branched profiles with an orthodox matrix organization. Lowercase labels identify grouped image sets.

**Figure 6:**
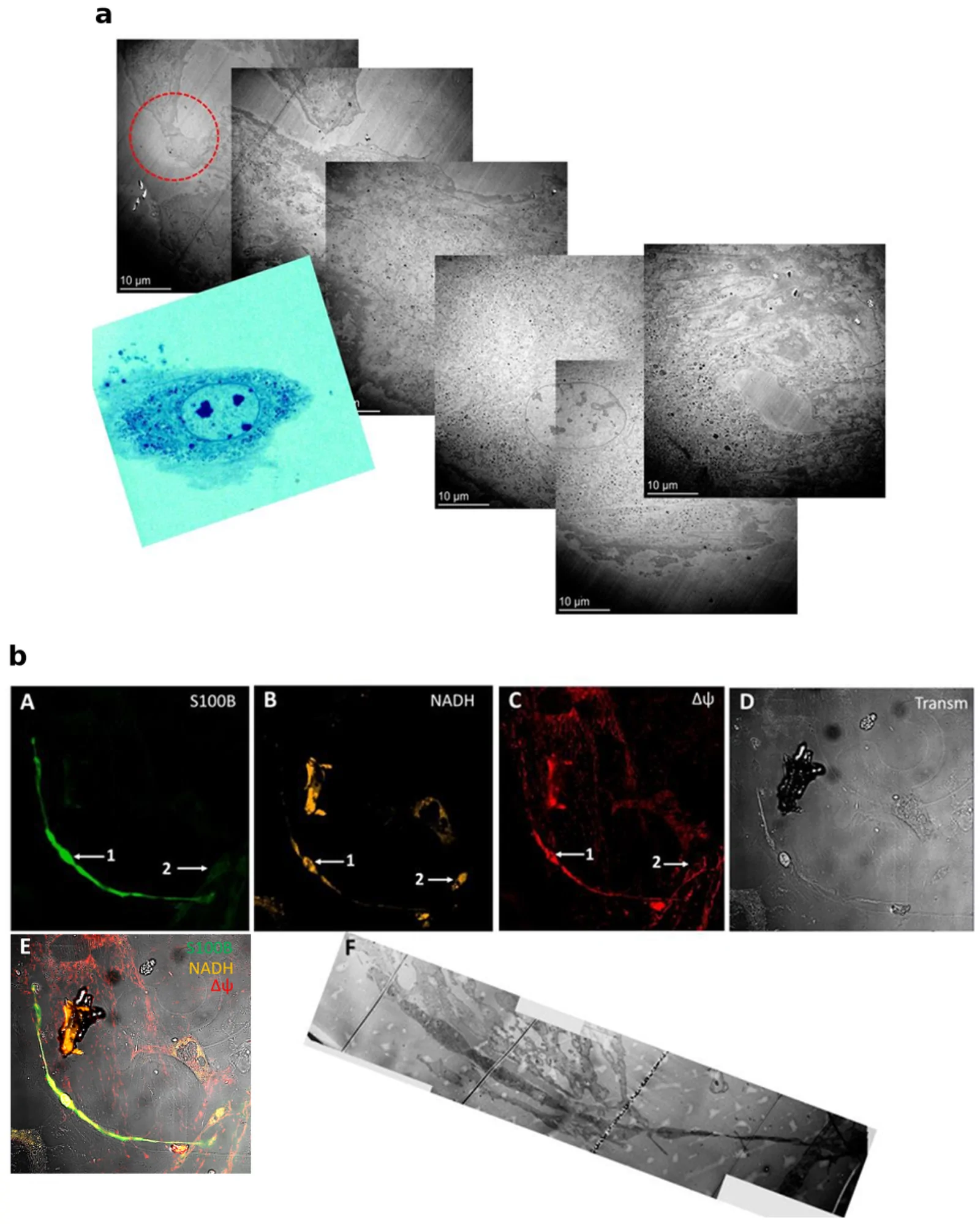
Selected ultrastructural comparison and elongated reporter-positive morphology. (**a**) Electron-microscopic reconstruction of a reporter-negative cell from the CLEM field (marked as 4) in Figure 4a. The broken red circle marks a close, gap-junction-like membrane contact with a neighboring reporter-positive cell; molecular junction identity was not tested. (**b**) A, shows strong EGFP fluorescence in the elongated cell; B, NAD(P)H autofluorescence; C, fluorescence of the Δψ-sensitive probe TMRM; D, transmitted light; E is the overlay of A–D; F is the ultramicroscopic reconstruction of the elongated SAN-derived S100B-EGFP+. 1,2 reporter-positive and negative cells, correspondingly. Lowercase labels identify grouped image sets; original arrows and scale bars are retained.

**Figure 7:**
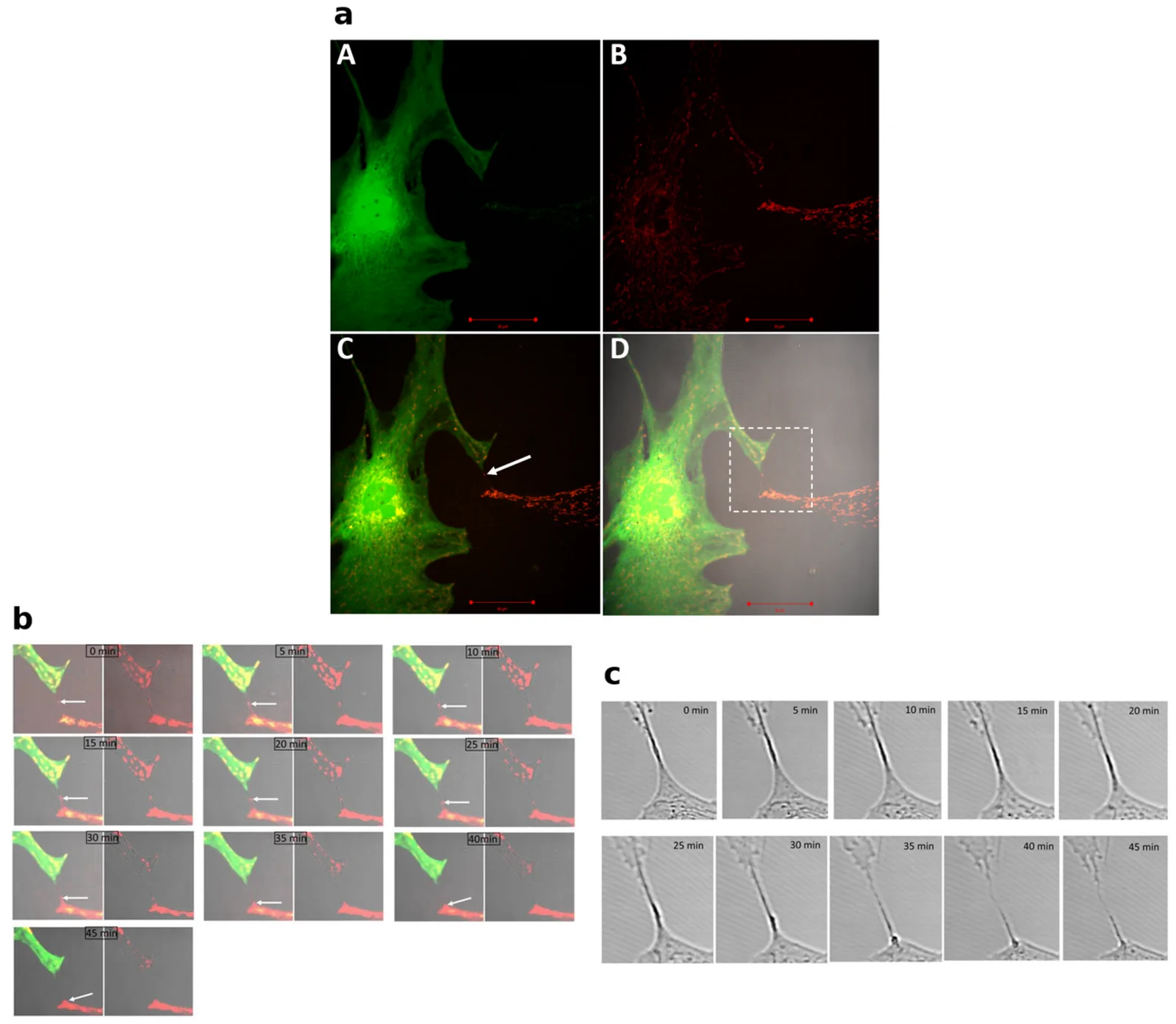
Single live-imaged movement event. (**a**) Baseline view of a thin connection between an S100B-EGFP+ cell and an EGFP− cell, with a TMRM-positive, mitochondrion-like structure in the center of the nanotube. A and B, EGFP, TMRM fluorescence correspondingly; C and D, overlay with dashed box of the region of interest (ROI); (**b**) Ten time points of ROI from 0 to 45 min show movement of the TMRM-positive structure toward the EGFP− cell; the white arrow follows the structure. Each time point includes an overlay and the TMRM channel alone. (**c**) Sequential transmitted-light images of the same nanotube, which begins to disassemble 35 min after the structure reaches the neighboring cell. Lowercase labels identify grouped image sets; original scale bars and annotations are retained. The full recording is provided as Supplementary Video S1.

**Figure 8:**
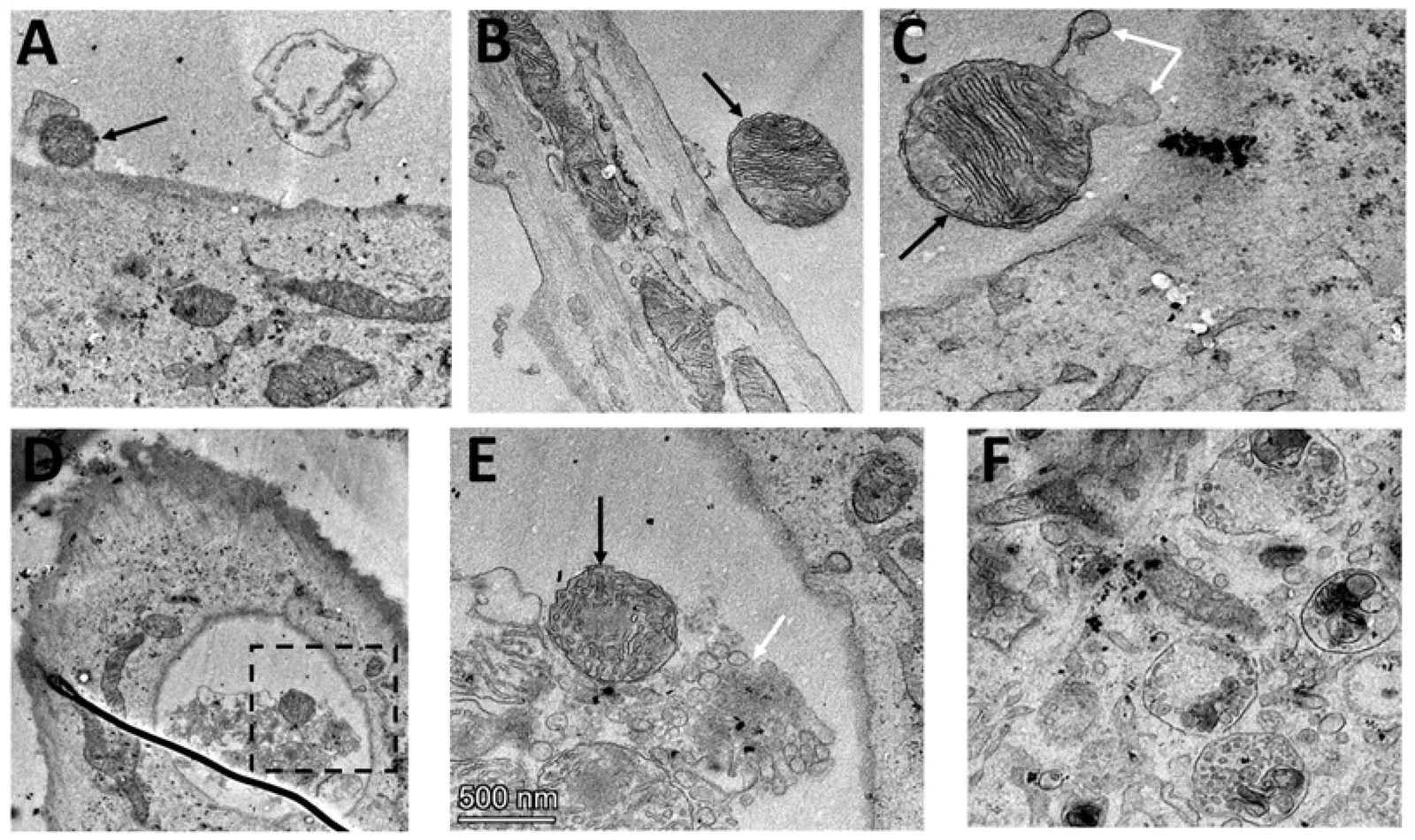
Additional static TEM observations from cultured preparations. Panel (**A**) shows a mitochondrial profile (black arrow) near and apparently attached to a cell surface. Panels (**B**,**C**) show mitochondrial-appearing profiles (black arrows) in extracellularly situated regions. White arrows show mitochondrial buds. Panels (**D**–**F**) show membrane-associated or cavity-like profiles containing mitochondria (black arrow) and vesicular structures (white arrow). These images were not reported as same-cell CLEM correlations; therefore, reporter status and cell identity are not established. The sections do not determine whether the profiles represent release, uptake, degradation, cell damage, or tangential sectioning, and they do not demonstrate mitochondrial function.

**Figure 9:**
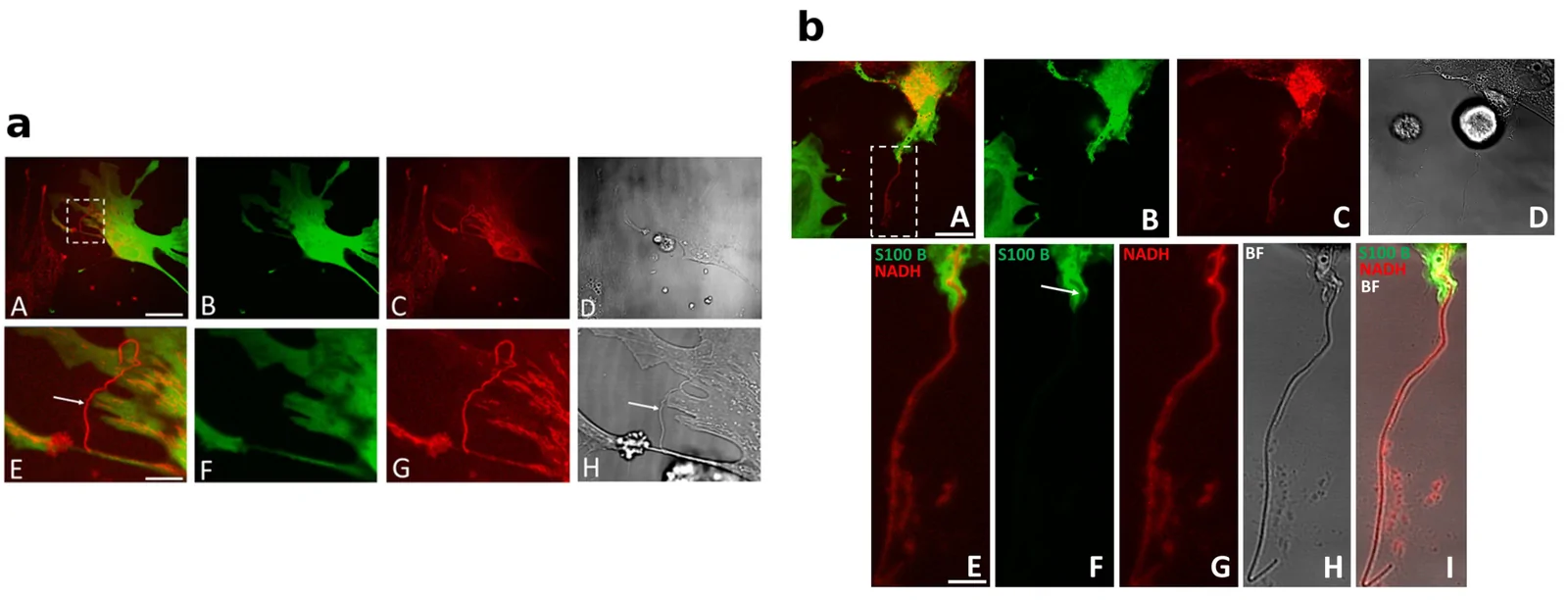
Elongated NAD(P)H-bright structures associated with S100B-EGFP+ cells. (**a**) Low- and high-magnification (A–D and E–H, correspondingly) views of an elongated fluorescent structure extending from a reporter-positive cell. These structures exhibit strong NAD(P)H autofluorescence (C and G) but lack S100B-associated EGFP signal (B and F), indicating selective transfer of mitochondrial material. A and E, overlay of NAD(P)H and EGFP. D and H, bright field. Bars, 50 µm in A–D and 10 µm in E–H. (**b**) A second field showing an elongated NAD(P)H-bright structure extending from a mitochondrial-rich region of a reporter-positive process. (A–D, low magnification (dashed white square is the region of interest (ROI)); E–K, high magnification of ROI confirms that mitochondrial filaments emerge from defined cellular regions densely populated with mitochondria (F, white arrow) but devoid of EGFP/S100B signal. Bars, 20 µm in A–D and 5 µm in E.

**Figure 10:**
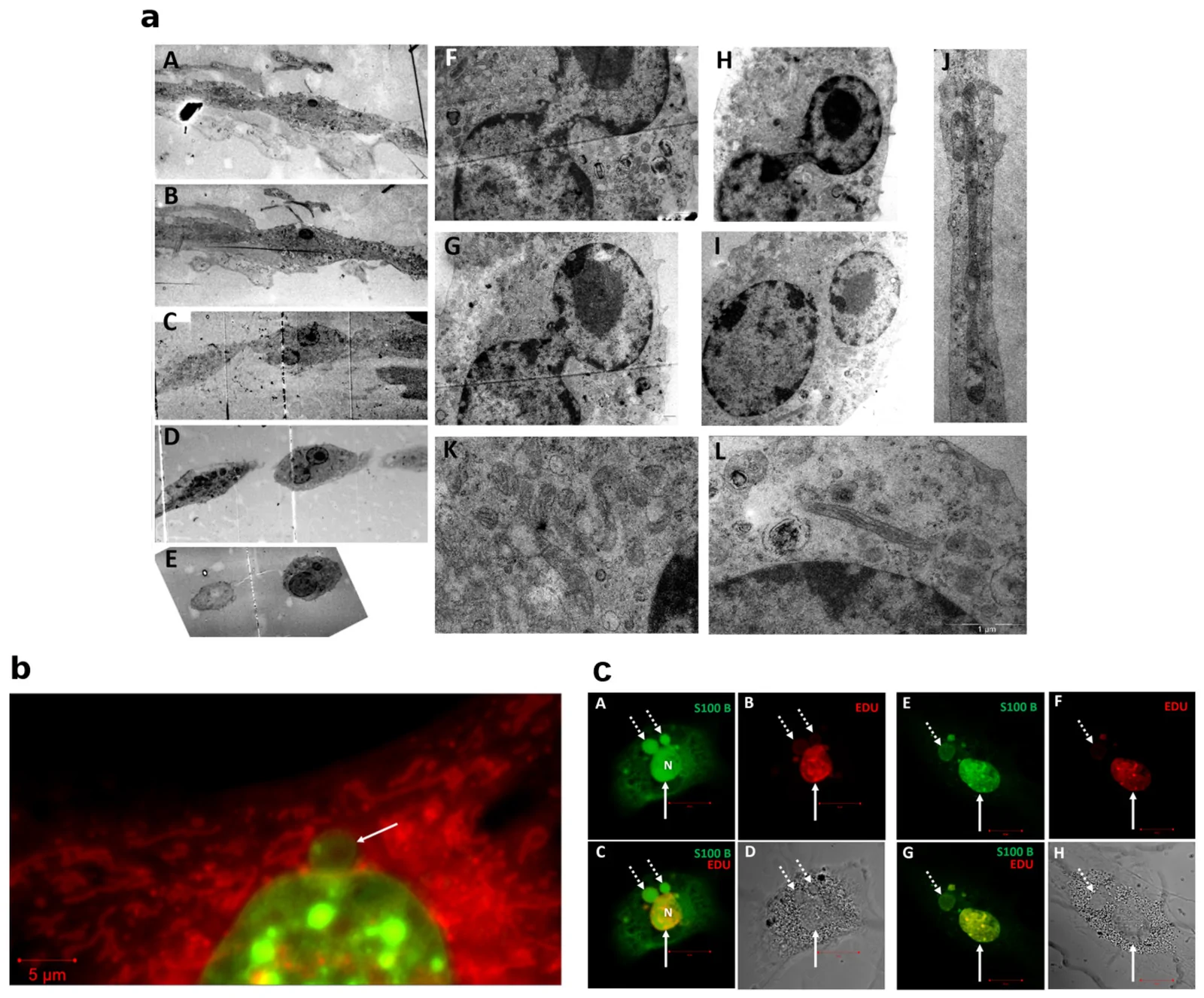
Nuclear heterogeneity in S100B-EGFP+ cells. (**a**) Selected serial electron micrographs show apparently separate nuclear profiles connected by a narrow bridge; A–E, five selected serial sections through the cell in the perinuclear zone. F–I, serial sections through the peri-nuclear zone of the cell at higher magnification, showing two nuclei connected by a narrow bridge. J, peripheral part of the cell containing a long mitochondrion with local lightening in the matrix; K, L the perinuclear region is enriched in mitochondria; (**b**) Confocal images of an EGFP+ cell with TMRM-stained mitochondria and NucBlue-stained nuclear material, including a small nuclear profile (bud) adjacent to the main nucleus. (**c**) Two reporter-positive cells with EdU fluorescence of different intensity among nuclear fragments are shown, one of which displays green fluorescence in both the nucleus (solid white arrow, N) and cytosol (A–D), while the other (E–H) shows strong EGFP fluorescence only in the nucleus. EdU staining (B and F) shows that not all nuclear fragments are equally positive for EdU. Broken arrows indicate faint staining of some fragments. D and H, transmittance. The images do not establish regulated nuclear budding, division, micronucleus timing, or whether EdU reflects replication or DNA repair.

**Figure 11:**
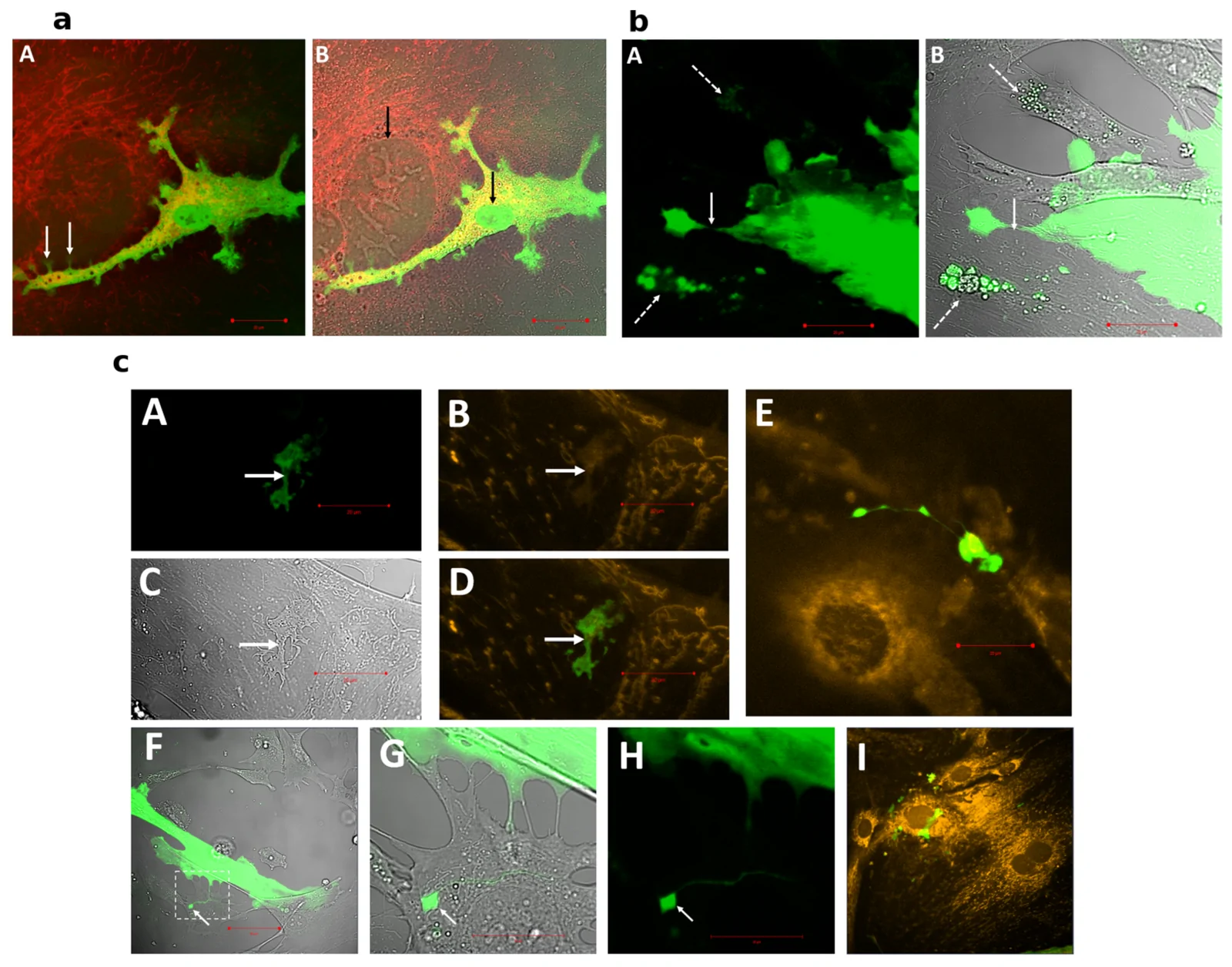
Reporter-positive cytoplasmic fragments and cell-overlying configurations. (**a**) An S100B-EGFP+ cell positioned over an EGFP− cell; arrows in the original image indicate nuclear profiles of different size (shown by black arrows) and sites of close apposition. Multiple adhesion sites (white arrows in A) are visible (mitochondria are stained with TMRM, red fluorescence). A, overlay of EGFP and TMRM fluorescence; B, as in A with transmittance to better resolve nuclear size. (**b**) Reporter-positive cytoplasmic material remains connected to a larger cell by a narrow bridge (shown by a solid white arrows), with nearby reporter-positive vesicular profiles (broken white arrows). A, EGFP fluorescence; B, overlay of EGFP and transmittance. (**c**) Detached reporter-positive fragments of varied morphology, some overlying neighboring cells and some containing detectable NAD(P)H-associated fluorescence. A–D, selected region of cultured cells: A, EGFP fluorescence; B, NAD(P)H fluorescence, demonstrating its low intensity in EGFP fragment; C, transmittance to better resolve spreading over EGFP-negative cell; D, overlay of EGFP and NAD(P)H; E–I, different examples to better demonstrate different morphology of fragments carrying EGFP. E and I, overlay of NAD(P)H and EGFP; F and G, overlay of EGFP and transmittance; H, EGFP fluorescence only taken from G. These images do not demonstrate selective targeting, uptake, contact-inhibition status, coordinated release, or intercellular signaling.

## Data Availability

The original contributions presented in this study are included in the article/Supplementary Material. Further inquiries can be directed to the corresponding authors.

## Supplementary Materials

The following supporting information can be downloaded at: https://www.mdpi.com/article/10.3390/cells15191762/s1, Video S1: Full time-lapse recording corresponding to Figure 7; Figure S1: Loss of mitochondrial-profile-associated TMRM fluorescence after mitochondrial de-energization with 5 µM nigericin.

## Abbreviations

AP	action potential
CLEM	correlative light and electron microscopy
EdU	5-ethynyl-2′-deoxyuridine
EGFP	enhanced green fluorescent protein
NAD(P)H	reduced nicotinamide adenine dinucleotide (phosphate)
SAN	sinoatrial node
TEM	transmission electron microscopy
TMRM	tetramethylrhodamine methyl ester
