# Supplementary material for: Correlative and Live-Cell Imaging of S100B-Promoter-Associated Interstitial Cells from the Cardiac Sinoatrial Node and Valves
Source: Cells. 2026 Sep 27;15(19):1762. doi: 10.3390/cells15191762 (PMC13663954; doi:10.3390/cells15191762)
Supplement: Supplementary file 1 [file cells-15-01762-s001.zip › Figure S1.pdf]

Supplementary Figure S1

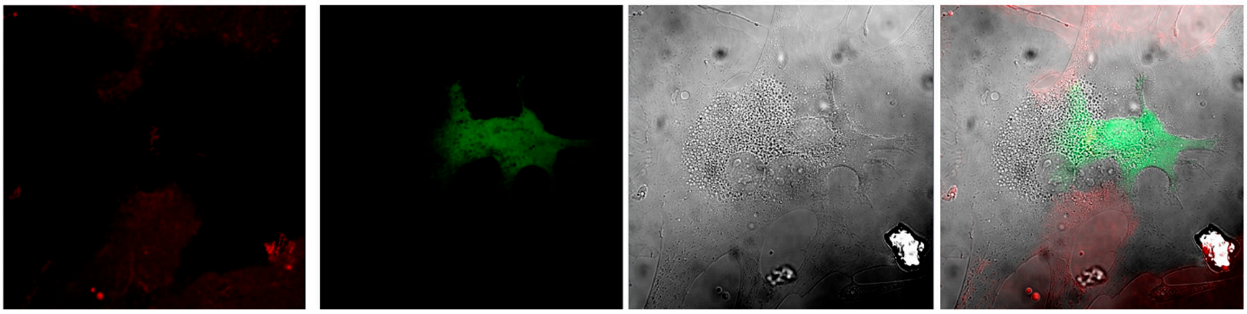

Supplementary Figure S1. Loss of mitochondrial-profile-associated TMRM fluorescence after mitochondrial de-energization with high concentration of nigericin. Cultured S100B-EGFP reporter cells were imaged after addition of 5 µM nigericin. From left to right, the panels show the TMRM channel, EGFP channel, transmitted light, and overlay. The red-channel gain was increased to visualize residual diffuse fluorescence. Mitochondrial-profile-associated TMRM fluorescence was no longer detectable in either the EGFP+ or EGFP- cell under these imaging conditions. This control demonstrates dye responsiveness to mitochondrial de-energization but does not calibrate absolute membrane potential.
